# Spectral Multi-Scale Attention Fusion Network for Rapid Detection of Black Tea Adulteration Using a Handheld Spectrometer

**DOI:** 10.3390/foods14244261

**Published:** 2025-12-10

**Authors:** Jiawei Tang, Yongyan Chen, Qing Meng, Bo Zhao, Dongling Qiao, Guohua Zhao, Jia Chen

**Affiliations:** 1 Chinese-Hungarian Cooperative Research Centre for Food Science, College of Food Science, Southwest University, Chongqing 400715, China; toukai3216@outlook.com (J.T.); cyy040215@outlook.com (Y.C.); qdttkl@163.com (D.Q.); 2 School of Westa, Southwest University, Chongqing 400715, China; 3 Integrative Science Center of Germplasm Creation in Western China (Chongqing) Science City, College of Food Science, Southwest University, Chongqing 400715, China; 4 Key Laboratory of Condiment Supervision Technology, State Administration for Market Regulation, Chongqing 400731, China; zhaobocq@163.com; 5 Chongqing Institute for Food and Drug Control, Chongqing 400731, China; 6 College of Life Sciences, Sichuan Normal University, Chengdu 610101, China; zhaoguohua1971@163.com

**Keywords:** black tea, artificial colorants, adulteration, handheld spectrometer, rapid detection

## Abstract

Black tea is a widely consumed beverage whose high economic value has led some producers to illegally add artificial colorants such as Sunset Yellow, Tartrazine, and Ponceau 4R, posing health risks. Although near-infrared (NIR) spectroscopy offers a rapid, non-destructive detection method, its use in trace-level colorant detection is limited due to low adulterant concentrations and interference from natural tea pigments. Hence, we developed a rapid, non-destructive method for detecting trace adulteration (from 0.1 to 0.5 g·kg^−1^) in black tea with artificial colorants using a handheld near-infrared spectrometer. To enhance sensitivity to low-level adulteration, we proposed a novel Spectral Multi-scale Attention Fusion Network (SMAFNet), designed to dynamically integrate multiscale features. SMAFNet consists of spectral preprocessing, multi-scale feature extraction, and cross-scale attention fusion modules. Comparative experiments with traditional machine-learning models demonstrated that SMAFNet achieved superior performance even at low adulteration levels. Sample sets (each including 36 samples) adulterated with Sunset Yellow, Tartrazine, and Ponceau 4R, SMAFNet achieved accuracies of 97.22–100%, F1-scores of 0.9879–1.00, and 100% recall. These findings confirm the feasibility and robustness of combining NIR with SMAFNet for the rapid and discriminative detection of trace colorants in black tea, offering a practical framework for on-site food safety monitoring and quality control.

## 1. Introduction

Black tea is one of the most widely consumed tea types worldwide and is renowned for its distinct flavor and bright liquor color. It is produced through a complete fermentation process, during which tea leaves undergo enzymatic oxidation, leading to the formation of characteristic pigments such as theaflavins and thearubigins [1,2,3]. In 2023, global tea production totaled 6.604 million tons, a 1.9% increase compared with 2022, with black tea representing more than 50% of the total production [4]. High-quality black tea infusions are bright and clear, whereas inferior products often appear dull or turbid. Owing to the high economic value of black tea, some producers illegally add artificial colorants such as Sunset Yellow, Tartrazine, and Ponceau 4R to conceal the inferior quality. These additives pose potential health risks and contravene food safety regulations. Excessive consumption of artificial colorants may lead to health risks, including genetic mutations, carcinogenesis, reduced hemoglobin levels, and allergic reactions [5]. Therefore, the detection of unauthorized colorant additives in black tea is essential to maintaining product integrity and safeguarding consumer safety.

Conventional analytical techniques for colorant detection include high-performance liquid chromatography [6], liquid chromatography–mass spectrometry [7], polarography [8], capillary electrophoresis [9], and thin-layer chromatography [10]. However, these methods are time consuming, instrument-intensive, and require skilled operators for data interpretation. Near-infrared (NIR) spectroscopy has emerged as a rapid, nondestructive analytical technique that requires minimal sample preparation and is environmentally friendly. When combined with chemometric algorithms, NIR has been successfully applied to adulteration detection [11,12], quality evaluation [13], geographical origin tracing [14], and process monitoring [15,16]. With technological advances and reduced equipment cost, handheld spectrometers have become suitable for fast, on-site analysis [17,18]. Their measurement range typically spans from 900 to 1700 nm [19]. However, only a few studies have investigated the detection of artificial colorants in teas using a handheld spectrometer, likely because of the low concentrations involved, as the quantitative detection limit of NIR is approximately 0.1% [20]. The amount of added colorant in black tea rarely exceeds 0.5 g·kg^−1^, as excessive addition darkens the infusion and degrades sensory quality. Furthermore, the presence of natural tea pigments, such as theaflavins and thearubigins, introduces spectral interference [21], complicating analysis. Consequently, a more efficient feature-extraction algorithm is required to obtain discriminative information from NIR.

Recently, one-dimensional convolutional neural networks (1D-CNNs) have shown strong capability for feature extraction from NIR data [22] and have been employed for quality assessment [23], component analysis [24,25], and adulteration or freshness detection [26,27]. However, traditional 1D-CNNs present certain limitations: the use of a single or fixed convolution kernel size restricts extraction to a specific scale [22], and the lack of cross-scale feature interaction hinders integration of multi-level spectral features, limiting sensitivity to weak signals from low-level adulteration.

To overcome these challenges, this study proposes a Spectral Multi-Scale Attention Fusion Network (SMAFNet), a novel spectral feature-extraction network that employs a multi-branch parallel architecture to simultaneously capture local and global spectral representations across multiple scales, thereby improving its ability to model complex spectral patterns. Thus, the study aims to develop an effective and non-destructive method for the rapid detection of trace synthetic colorant adulteration in black tea, thereby supporting on-site food safety monitoring and quality control.

## 2. Materials and Methods

### 2.1. Sample Preparation and Spectra Acquisition

Black tea samples were obtained from Xida Tea Co., Ltd. (Chongqing, China). Food-grade colorants, including Tartrazine, Sunset Yellow, and Ponceau 4R, were purchased from Kuoyi Biotechnology Co., Ltd. (Jinan, China). To minimize light-scattering effects caused by particle-size variation and to ensure spectral uniformity, all black tea samples were ground and sieved through a 60-mesh screen, sealed in airtight polyethylene bags, and stored in a cold, dry environment until analysis. For the adulterated samples, black tea powder was homogeneously mixed with one of the three colorants: Tartrazine, Sunset Yellow, or Ponceau 4R. The added colorant concentration ranged from 0.1 to 0.5 g·kg^−1^, at 0.05 g·kg^−1^ intervals. Before spectral measurement, the moisture content of all samples was monitored and adjusted as necessary to maintain consistency. The final moisture content of black tea samples was maintained at approximately 6.9% to 7.2%, with no significant differences among them.

Spectral data were collected using a handheld spectrometer (model NIR-R210, Shenzhen Pynect Science and Technology Co., Ltd., Shenzhen, China) operating over the 900–1700 nm range with a spectral resolution of 3 nm. To ensure measurement stability, the spectrometer was preheated for 3 min before use, and the ambient temperature was maintained as constant as possible during acquisition. Each sample was scanned six times, and the mean spectrum was used for analysis. In total, 345 spectra were obtained, including 45 pure-tea and 300 adulterated samples (100 per colorant).

### 2.2. Dataset Partition

To develop and evaluate the predictive model, the tea samples were divided into calibration and validation subsets. The SPXY algorithm [28] was employed to split each dataset, with 75% of the samples allocated for training and the remaining 25% reserved for validation. The SPXY ensures spatial uniformity by considering both spectral variables (X) and reference values (Y), rendering it particularly suitable for partitioning low-concentration datasets. Once a sample is assigned to the training set by SPXY, it is exclusively retained in that set, preventing any overlap between the two subsets. This guarantees that the model is evaluated on truly unseen data.

### 2.3. Construction of SMAFNet

**Figure 1:**
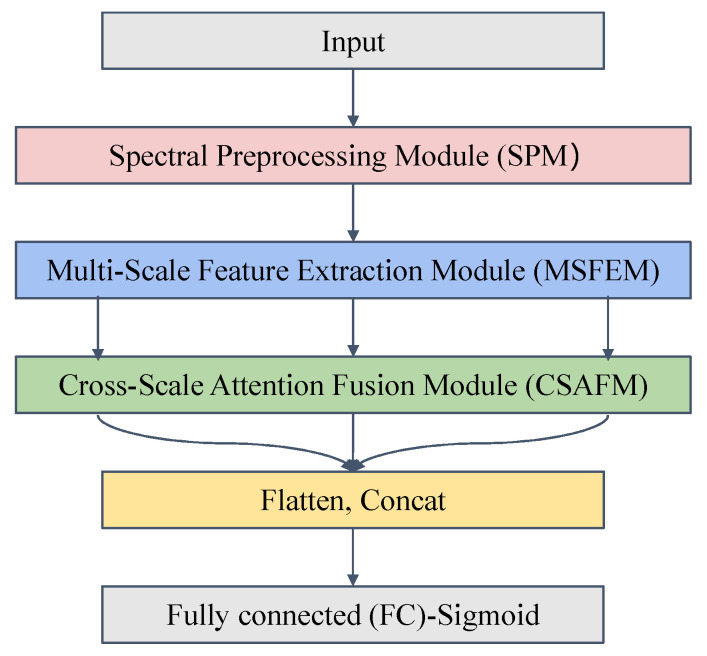
Architecture of the proposed spectral multi-scale attention fusion network (SMAFNet).

The overall architecture of SMAFNet (Figure 1) comprises three functional modules: (1) a Spectral Preprocessing Module (SPM), designed to enhance input consistency and suppress noise; (2) a Multi-Scale Feature Extraction Module (MSFEM), which captures hierarchical spectral characteristics across different receptive fields; and (3) a Cross-Scale Attention Fusion Module (CSAFM), which dynamically integrates multi-scale features to enhance the model’s sensitivity to trace adulterants.

Following feature fusion, the resulting features were flattened, concatenated, and passed through fully connected layers for the final classification output.

#### 2.3.1. Spectral Preprocessing Module

The SPM was developed to preprocess raw 1D spectral data with the dual objectives of suppressing instrumental noise and adjusting feature dimensions, thereby establishing a stable foundation for downstream feature extraction.

**Figure 2:**
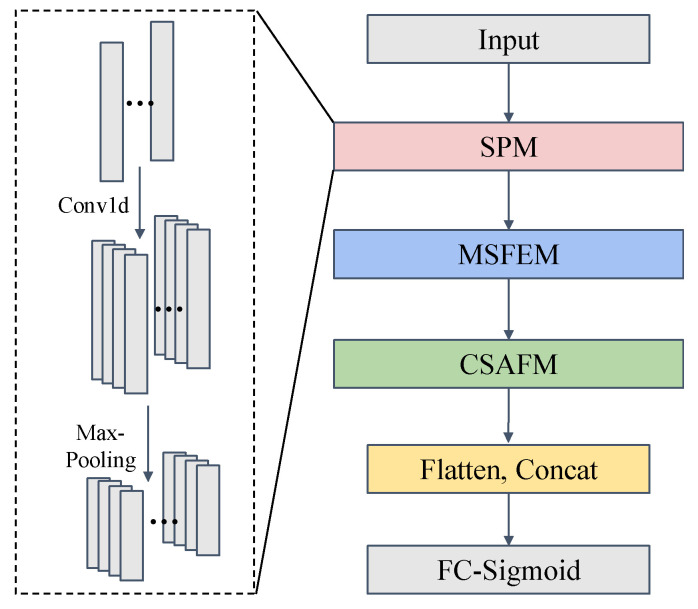
Structure of the spectral preprocessing module (SPM).

It comprises a 1D convolutional (Conv1d) layer and a max-pooling layer (Figure 2). The Conv1d layer performs initial spectral feature extraction and channel-dimension adjustment, whereas the max-pooling layer executes dimensionality reduction.

Conv1d layer: This layer applies a set of learnable convolution kernels to the raw spectral input to implement preliminary feature extraction and channel-dimension adjustment. For a raw spectral input $X=\;\left[x_{1},x_{2},\ldots ,x_{n}\right]$, where $X\;\in \;R^{C_{i}\times L}$, $C_{i}$ denotes the initial input channel count (initial = 1), and *L* represents the number of spectral features. The Conv1d layer employs convolution kernels $W=\;\left[w_{1},w_{2},\ldots ,w_{C_{o}}\right]$, where $W\;\in \;R^{k\times C_{i}\times C_{o}}$, *k* is the kernel size and *C*_0_ is the number of output channels. For the *j*-th output channel, the value at position *t* in the feature map *y*^(*j*)^ is computed as

$$y^{\left(j\right)}=\sum_{i=0}^{k-1}x_{t+i}\cdot W_{i}^{\left(j\right)}\;\left(1\;\leq \;t\;\leq \;n\;-\;k+1\right)$$

Max-pooling layer: This layer performs dimensionality reduction on the Conv1d output, compressing redundant information while preserving key discriminative spectral features.

#### 2.3.2. Multi-Scale Feature Extraction Module

Based on the Inception module in GoogLeNet [29], the MSFEM was designed to overcome the limitations of traditional 1D-CNNs. The MSFEM extracts spectral features across multiple receptive fields, thereby enhancing the ability of the model to identify weak signals from low-level adulterants.

**Figure 3:**
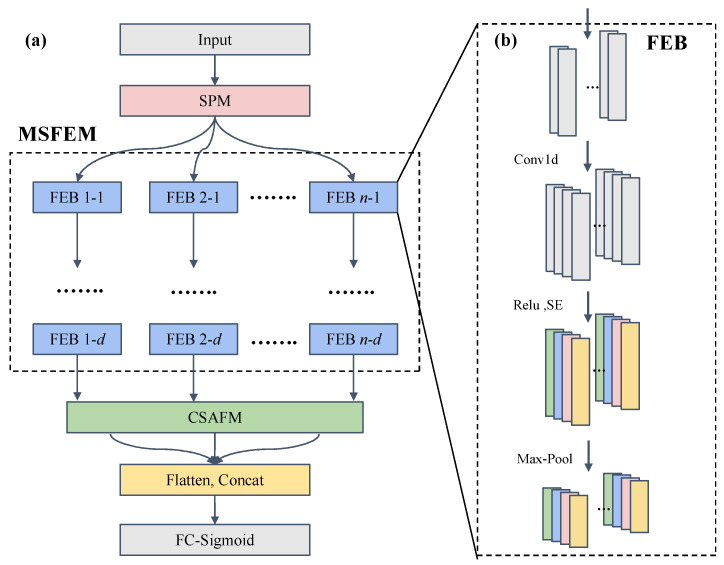
(**a**) Architecture of the multi-scale feature extraction module (MSFEM). (**b**) Structure of a single feature extraction block (FEB).

It comprises a set of *n* parallel feature extraction blocks (FEBs), each employing Conv1d kernels of different sizes (Figure 3a). In this study, *n* was set between 1 and 3, and six kernel sizes were tested: [1 × 1], [3 × 1], [5 × 1], [7 × 1], [9 × 1], and [11 × 1]. The MSFEM also features a configurable network depth *d* (ranging from 1 to 3), defined as the number of sequentially stacked FEBs applied to the spectral representations.

Subsequent experiments systematically investigated the effects of the kernel size, scale number (*n*), and depth (*d*) on the MSFEM feature extraction performance.

A single FEB (Figure 3b) performs four operations:

1. Convolution: A Conv1d layer with a specific kernel size extracts spectral features corresponding to its receptive field.
2. Activation: A rectified linear unit (ReLU) introduces a nonlinearity feature extraction.
3. Channel recalibration: A squeeze-and-excitation (SE) module enhances the model’s representational capacity by emphasizing informative channels while suppressing less relevant channels [30]. The SE module operates in three stages: (i) Squeeze: Global average pooling is applied to the input feature map to compress the spectral information of each channel into a single scalar; (ii) Excitation: Two fully connected layers, followed by ReLU and Sigmoid activations, are used to learn the relative importance of each channel; and (iii) Reweighting: The learned attention weights are applied to the original feature map through channel-wise multiplication.
4. Pooling: A max-pooling layer reduces feature dimensionality.

#### 2.3.3. Cross-Scale Attention Fusion Module

**Figure 4:**
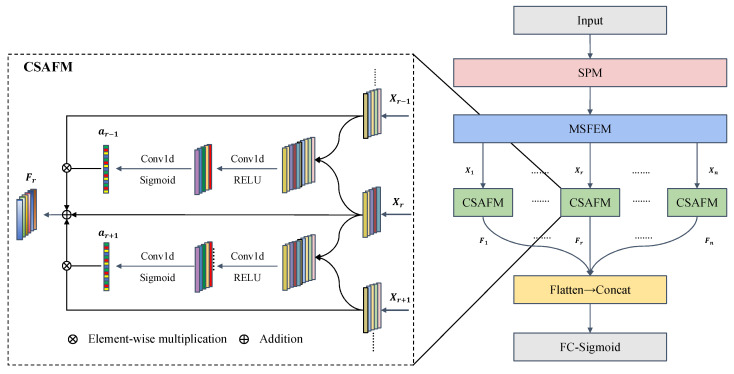
Structure of the cross-scale attention fusion module (CSAFM).

The CSAFM was designed by integrating the key mechanisms of the SE network and the bidirectional feature pyramid network (BiFPN) [31], with the objective of enhancing feature interaction and selective aggregation across scales. The CSAFM comprises two Conv1d layers and two activation functions (ReLU and Sigmoid), and its workflow involves three main stages (Figure 4):

1. Cross-scale interaction

For each reference-scale feature $X_{r}$, where *r* denotes the reference scale, the module sequentially fuses $X_{r}$ with features from all other scales ($X_{j}$, *j* ≠ *r*). First, $X_{r}$ and $X_{j}$ are concatenated along the channel dimension to construct a cross-scale interaction representation $Z_{j}$:

(1) $$\begin{array}{c} Z_{j}\;=\;Concat\left(X_{r},X_{j}\right) \end{array}$$

2. Attention-weight generation and feature recalibration

The concatenated feature $Z_{j}$ is fed into a Conv1d layer (Conv1d_1_) and activated by ReLU to extract the fused feature representations. A second Conv1d layer (Conv1d_2_) and Sigmoid activation are then applied to generate attention weights $a_{j}$:

(2) $$\begin{array}{c} a_{j}\;=\;\sigma \left({\mathrm{Conv}1d}_{2}\left(\mathrm{RELU}\left({\mathrm{Conv}1d}_{1}\left(Z_{j}\right)\right)\right)\right) \end{array}$$

where $\sigma \left(\cdot \right)$ denotes the Sigmoid function, mapping the weights to the range (0, 1). The generated weights $a_{j}$ are multiplied element-wise with the corresponding scale feature $X_{j}$ along the channel axis to achieve adaptive recalibration:

(3) $$\hat{X_{j}}\;=\;a_{j}\;⨀{\;X}_{j}$$

where ⊙ represents element-wise multiplication.

3. Feature aggregation

The recalibrated features from all the scales ($\hat{X_{j}}$) are added to the reference feature $X_{r}$ to obtain the final fused feature $F_{r}$:

(4) $$\begin{array}{c} F_{r}\;=\;X_{r}\;+\;\sum_{j\neq r}\hat{X_{j}} \end{array}$$

After repeating this process for all scales, the fused features were flattened, concatenated, and passed into fully connected layers for classification.

### 2.4. Model Transmission

The structural parameters of SMAFNet were first optimized using a dataset containing pure samples and samples adulterated with Sunset Yellow. Sunset Yellow is a widely used artificial colorant with a low acceptable daily intake (ADI) value [5], indicating higher toxicity and thus requiring priority in detection. The optimized SMAFNet was then transferred to the datasets containing pure and Tartrazine- or Ponceau 4R-adulterated samples to evaluate its generalization performance in multitype adulteration detection. Its cross-domain adaptability was further validated by transferring SMAFNet to an open-source tablet NIR dataset (310 samples, available at http://www.models.life.ku.dk/, accessed on 8 May 2025). The NIR spectra of tablet samples are plotted in Supplementary Figure S1, and the validation results are provided in Supplementary Table S1 and Figure S2.

### 2.5. Model Evaluation

The performance of SMAFNet was evaluated by conducting comparative experiments between SMAFNet and several traditional machine learning models: partial least squares-discriminant analysis (PLS-DA), radial basis function support vector machine (RBF-SVM), random forest (RF), 1D-CNN, and multilayer perceptron (MLP). The hyperparameters of PLS-DA, RBV-SVM, and RF were optimized using a grid search, while 1D-CNN, MLP, and SMAFNet were trained using the adaptive moment estimation (Adam) optimizer [32] with an initial learning rate of 0.001.

Model performance was assessed using four metrics: accuracy (ACC), precision (PRE), recall (REC), and F1-score. Their definitions are expressed as follows:

(5) $$\mathrm{ACC}\;=\;\frac{\mathrm{TP}\;+\;\mathrm{TN}}{\mathrm{TP}\;+\;\mathrm{TN}\;+\;\mathrm{FP}\;+\;\mathrm{FN}}\times 100\%$$

(6) $$\mathrm{PRE}=\frac{\mathrm{TP}}{\mathrm{TP}+\mathrm{FP}}\times 100\%$$

(7) $$\mathrm{REC}=\frac{\mathrm{TP}}{\mathrm{TP}+\mathrm{FN}}\times 100\%$$

(8) $$F\text{1-score}=2\;\times \;\;\frac{\mathrm{PRE}\;\times \;\mathrm{REC}}{\mathrm{PRE}+\mathrm{REC}}$$

In this study, TP (true positive) is the number of adulterated samples correctly identified as “adulterated”; TN (true negative) is the number of pure samples correctly classified as “unadulterated”; FP (false positive) is the number of pure samples incorrectly labeled as “adulterated”; and FN (false negative) is the number of adulterated samples incorrectly classified as “unadulterated.”

## 3. Results and Discussion

### 3.1. Spectra Analysis

**Figure 5:**
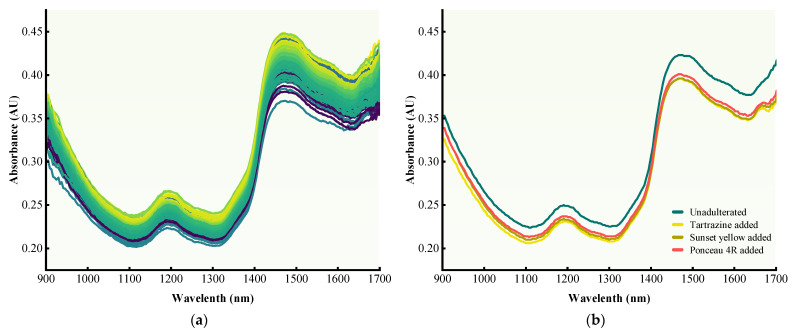
(**a**) Near-infrared (NIR) spectra of all samples; (**b**) average spectra of unadulterated and colorant-adulterated samples.

The original NIR spectra of the experimental samples are presented in Figure 5a. The spectral curves of all samples were highly similar, making it challenging to distinguish adulterated from pure samples by visual inspection alone. All samples exhibited weak absorption bands in the range of 1170–1220 nm, along with distinct absorption peaks in the range of 1430–1500 nm and 1680–1700 nm. A consistent spectral trend was observed across all specimens tested. The absorption peak around 1195 nm may originate from the second overtone stretch vibration of C–H and O–H [33]. The spectral band at roughly 1465 nm may derive from the O–H first overtone vibrations [33]. The absorption bands near 1656 and 1680 nm were correlated with catechin content, primarily associated with the C–H and S–H first overtone vibrations [34]. In addition, the spectral patterns observed in this study are consistent with those previously reported for fermented tea [33]—a variety closely related to black tea. This similarity in spectral profiles may be attributed to analogous biochemical transformations occurring during the fermentation.

The characteristics of the different samples were explored by computing and plotting the mean spectra of adulterated and unadulterated black tea samples (Figure 5b). The results revealed a slight decrease in absorbance following the addition of colorants, suggesting that these artificial additives exert a measurable influence on the NIR spectra. However, owing to spectral noise and other interfering factors, substantial spectral overlap remained among the samples.

### 3.2. Dataset Division

All samples were divided into calibration and validation subsets using the SPXY algorithm, and three binary classification datasets were constructed (Table 1). Each dataset comprised unadulterated samples and samples adulterated with one of the three colorants. The differences among the datasets are attributed to the inherent mechanisms of the SPXY algorithm, which selects representative samples by maximizing diversity in both the spectral (X) and property (Y) spaces, where Y denotes the adulteration status. Because of the spectral responses of Tartrazine, Sunset Yellow, and Ponceau 4R, their interactions with the SPXY selection logic led to slight variations in the number of pure samples assigned to each subset. Overall, each dataset contained 109 calibration samples and 36 validation samples.

### 3.3. Construction and Parameter Selection of SMAFNet

SMAFNet was optimized using the Sunset Yellow-adulterated dataset for both module configuration and parameter selection. The cross-entropy loss function was adopted as the objective function, and an Adam optimizer was employed with an initial learning rate of 0.001, beta1 = 0.9, beta2 = 0.999, and epsilon = 10^−8^ was employed. Model performance was evaluated using two metrics: ACC and F1-score.

#### 3.3.1. SPM Construction

The SPM was introduced to suppress noise and enhance the quality of input features. Three kernel sizes, each coupled with different output-channel configurations, were evaluated (Table 2). The performance of the SPM was strongly influenced by both the kernel size and the number of output channels in the convolutional layer. The medium-sized kernel [16 × 1] achieved the highest accuracy (71.56%) and F1-score (0.7737) compared with the other configurations. This behavior can be explained by the fact that larger kernels tend to overlook fine spectral variations, whereas smaller kernels may fail to capture global characteristics. Regarding the number of output channels, the results indicated that configurations with 64 channels generally yielded comparable or slightly superior performance to those with 128 channels across all kernel sizes. A max-pooling layer with 64 channels in the SPM was sufficient to reduce the dimensionality of the input spectral sequence, compress redundant information, and preserve discriminative features. Therefore, the [16 × 1] kernel coupled with 64 output channels was selected as the optimal SMP configuration for subsequent experiments.

#### 3.3.2. MSFEM Construction

The selection of kernel sizes and the number of scales directly influence the sensitivity of MSFEM to features at different resolutions, whereas network depth affects the complexity of feature extraction. Accordingly, the MSFEM was constructed and optimized by sequentially evaluating the kernel size, scale number, and network depth.

##### Kernel Size and Scale Selection

The effects of convolutional kernel size and scale diversity on the feature-extraction capability of the MSFEM were investigated by testing convolutional kernels ranging from [1 × 1] to [11 × 1] (in odd-numbered increments) under single-, dual-, and tri-scale configurations (Table 3).

In the single-scale experiments, the [5 × 1] kernel achieved the best performance, with an ACC of 79.82% and an F1-score of 0.8472. This advantage stemmed from its balanced receptive field, which effectively captured both fine local spectral details and medium-range spectral trends. The [1 × 1] kernel was excluded from single-scale testing because of its ultranarrow receptive field, which was insufficient to capture effective spectral correlations on its own.

For dual-scale configurations, all kernel pairings outperformed the best single-scale kernel [5 × 1]. Pairings of small-to-medium or small-to-large kernels demonstrated superior performance: for example, the [3 × 1, 5 × 1] pairing reached an ACC of 85.32%; the [1 × 1, 9 × 1] pairing achieved the same ACC. This improvement stemmed from their complementary receptive-field coverage—the [1 × 1] kernel effectively extracted local spectral details, whereas the [3 × 1] and [5 × 1] kernels captured medium-range spectral correlations, together enabling the robust identification of weak adulteration signals. In contrast, pairings of medium-to-large or large-to-extra-large kernels (e.g., [9 × 1, 11 × 1]) exhibited lower efficiency because overlapping global receptive fields introduced information redundancy.

In the tri-scale experiments, dispersed kernel sets outperformed adjacent kernel sets. Among all configurations, the [1 × 1, 5 × 1, 9 × 1] combination achieved the highest ACC (92.66%) and F1-score (0.9452). This configuration encompassed three distinct receptive field scales: the [1 × 1] kernel captured fine local details, the [5 × 1] kernel extracted medium-range correlations, and the [9 × 1] kernel captured global spectral trends. This multiscale coverage minimized receptive field overlap and fully extracted hierarchical spectral information, which is crucial for detecting trace-level adulteration.

Based on these results, the MSFEM was optimized to a tri-scale configuration using [1 × 1, 5 × 1, and 9 × 1] kernels, providing a robust feature-extraction foundation for the subsequent CSAFM. Notably, the instances of lower accuracy observed during the initial stages of hyperparameter optimization for individual modules do not reflect the performance of the final, fully optimized SMAFNet model. These lower accuracies only reflect the performance of sub-optimal configurations or isolated modules prior to full integration and fine-tuning of the complete architecture.

##### Network Depth Selection

To further optimize the MSFEM, the network depth (*d*) was set to 1, 2, and 3 for comparative experiments (Table 4).

As shown in the table, increasing the depth (*d*) of the MSFEM initially improved model performance, but eventually caused a decline. When *d* was increased from 1 to 2, the ACC improved from 92.66% to 95.41%, and the F1-score increased from 0.9452 to 0.9660, demonstrating that moderate depth enhanced feature abstraction and representation. This improvement can be attributed to a moderately deepened network, which strengthens the model’s ability to extract hierarchical spectral features by integrating local, medium-range, and global information for better detection of weak adulteration signals. However, when the depth was further increased to *d* = 3, model performance declined, as the ACC and F1-score decreased to 94.50% and 0.9595, respectively. This suggests that excessive depth introduces overfitting and reduces the generalization capability of the model. Based on these results, the optimal network depth for the MSFEM was determined to be *d* = 2.

#### 3.3.3. Architecture of SMAFNet

After the aforementioned processing steps, the CSAFM was used to integrate information from multiple scales. Accordingly, the processing workflow of the SMAFNet can be described as follows:

The raw spectra were first preprocessed and dimensionally reduced by the SPM, which adopted the optimized configuration of a [16 × 1] convolutional kernel with 64 output channels. Subsequently, the MSFEM—configured as a tri-scale structure with convolutional kernels of [1 × 1], [5 × 1], and [9 × 1], and a network depth of *d* = 2—extracted multiscale spectral features from the preprocessed data. The CSAFM then integrated features from different scales through cross-scale interaction and attention-based recalibration. After flattening and concatenation, the fused features were fed into the fully connected layer, where a Sigmoid activation function was applied to complete the classification task.

**Figure 6:**
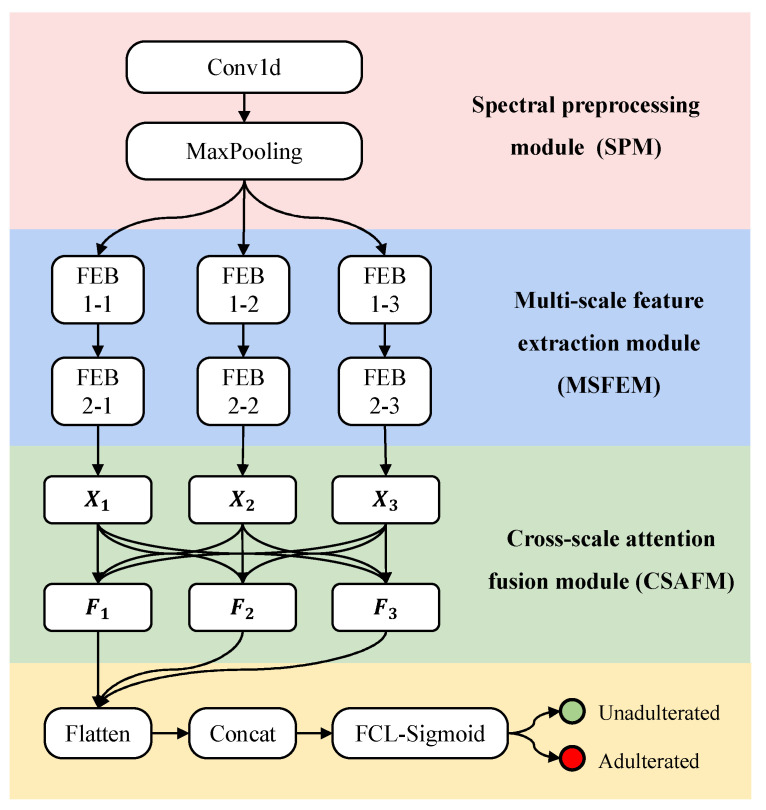
Final architecture of SMAFNet.

The final architecture of SMAFNet is illustrated in Figure 6; the detailed model parameters are provided in Supplementary Table S1.

### 3.4. Validation and Performance Comparison

To evaluate the performance of the optimized SMAFNet in identifying trace levels of artificial colorant adulteration in black tea, several traditional machine-learning models were implemented and validated under consistent experimental conditions, including PLS-DA, RBF-SVM, RF, 1D-CNN, and MLP. The final performance metrics of each model on the Sunset Yellow-adulterated dataset is listed in Table 5.

As shown in Table 5, the RBF-SVM—which achieved the best performance among the traditional models—obtained an ACC of 86.11% and an F1-score of 0.9123. Previous studies have demonstrated the nonlinearity of tea spectra obtained using portable spectrometers [19], which explains why the RBF-SVM performed better than the other traditional models. However, the selection of kernel parameters in an SVM lacks a universally effective strategy and typically depends on grid or random search within a predefined parameter space. These approaches are computationally intensive and may yield suboptimal performance owing to limited exploration efficiency [35]. PLS-DA exhibited the lowest ACC (72.22%), as linear models cannot resolve the nonlinear spectral interference caused by artificial colorants [36].

**Figure 7:**
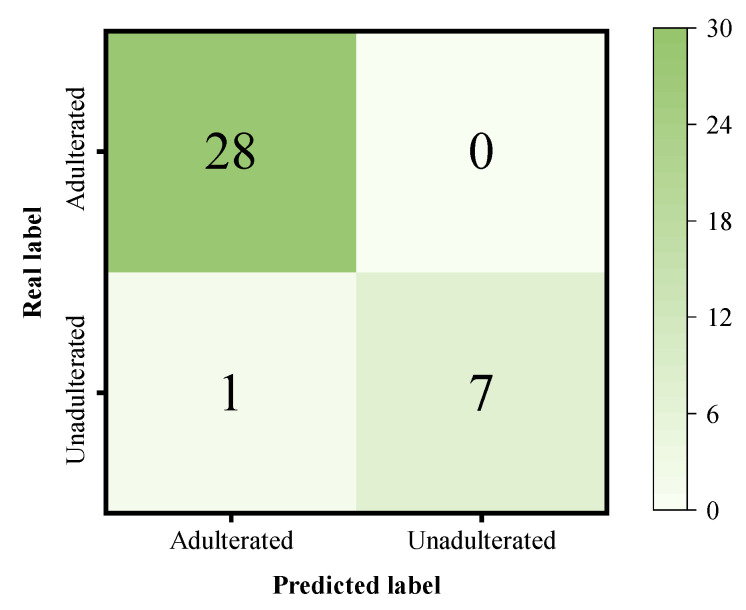
Confusion matrix of SMAFNet for the discrimination of Sunset Yellow-adulterated black tea.

In contrast, SMAFNet outperformed all other models across all evaluation metrics, achieving an ACC of 97.22%, REC of 100%, and F1-score of 0.9825. The confusion matrix of SMAFNet for Sunset Yellow adulteration prediction on the validation set is shown in Figure 7. This superior performance arises from the synergy among its modular components: the SPM suppresses spectral noise while preserving key feature information; the MSFEM effectively captures spectral characteristics ranging from local details to global trends and enhances salient channel information through the SE module; and the CSAFM mitigates information barriers between multi-level features, thereby maximizing the retention of weak spectral signals from trace colorants. Although one unadulterated sample was misclassified as adulterated, resulting in a slight decrease in accuracy, the model successfully identified all adulterated samples, achieving 100% recall. From a food safety perspective, such conservative misclassification is acceptable. These results demonstrate that SMAFNet possesses excellent discriminative capability for the detection of Sunset Yellow adulteration in black tea.

### 3.5. Transfer Performance of SMAFNet

To further evaluate the generalization and transferability of the optimized SMAFNet, the optimized network was transferred to Tartrazine- and Ponceau 4R-adulterated datasets to validate its detection performance. For comparison, PLS-DA, RBF-SVM, RF, 1D-CNN, and MLP models were also trained and tested on these two datasets under identical conditions.

**Figure 8:**
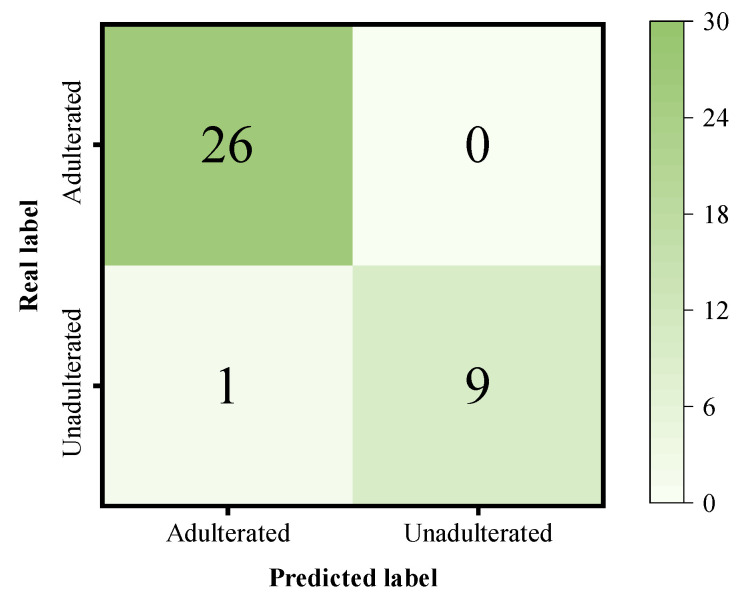
Confusion matrix of SMAFNet for the discrimination of Tartrazine-adulterated black tea.

For the Tartrazine-adulterated dataset (Table 6), SMAFNet maintained the highest performance, achieving an ACC of 97.22%, an REC of 100%, and an F1-score of 0.9811. The discrimination confusion matrix of SMAFNet for Tartrazine adulteration is shown in Figure 8. Among the traditional models, RBF-SVM performed best, with an ACC of 86.11% and an F1-score of 0.9123, but still showed a significant gap when compared with SMAFNet. In contrast, PLS-DA exhibited the lowest performance (ACC = 75.00%), underscoring the limitations of linear models in capturing the complex nonlinear relationships within spectral data.

**Figure 9:**
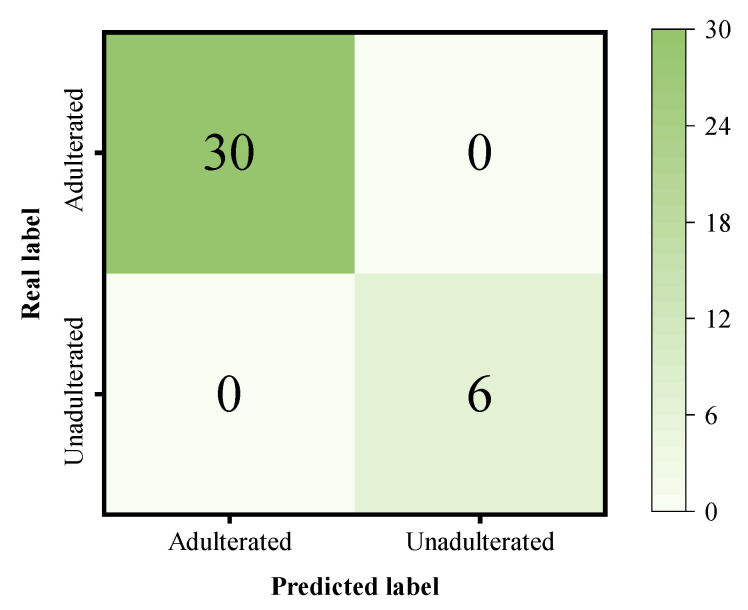
Confusion matrix of SMAFNet for the discrimination of Ponceau 4R-adulterated black tea.

For the Ponceau 4R-adulterated dataset (Table 7), SMAFNet achieved perfect results across all metrics (ACC = 100%, PRE = 100%, REC = 100%, F1-score = 1.0), demonstrating complete discriminative capability. The best-performing baseline, RF, attained only an ACC of 86.11% and an F1-score of 0.9123, whereas the RBF-SVM, which had performed well in the Sunset Yellow dataset, exhibited a marked decline (ACC = 83.33%, F1-score = 0.8929). These findings confirm that SMAFNet not only provides a strong discriminative power for single-colorant adulteration but also demonstrates excellent transferability across different adulterant types. The confusion matrix of SMAFNet for Ponceau 4R-adulterated samples is shown in Figure 9.

The transfer performances across different types of artificial colorants demonstrated that SMAFNet’s modular design provides strong adaptability. Although this study focused on artificial colorants in black tea, the underlying principles of spectral analysis and deep learning are broadly applicable. SMAFNet can be extended to detect other types of adulterants or contaminants in various food products, provided that appropriate spectral data for these substances are available for training. However, additional studies are required to validate its generalization across diverse food matrices and adulterant types.

## 4. Conclusions

To address the challenges of weak spectral signals and limited feature separability in detecting trace levels of artificial colorant adulteration in black tea, this study proposed and validated a novel SMAFNet comprising three synergistic modules. Compared with traditional machine-learning models, SMAFNet achieved superior performance, with ACC ranging from 97.22% to 100%, F1-scores between 0.9879 and 1.00 and perfect recall (100%) for all adulterated samples (Sunset Yellow, Tartrazine, and Ponceau 4R), ensuring zero missed detections. This high sensitivity is critical for reliable food safety monitoring. When combined with a handheld NIR device, SMAFNet allows for quick analysis and provides near real-time results, offering a practical framework for on-site food safety monitoring.

SMAFNet’s superior performance can be attributed to its cross-scale attention fusion mechanism. By dynamically weighting and integrating multiscale features, SMAFNet effectively mitigated interference from the intrinsic chromogenic compounds of black tea, such as theaflavins and thearubigins, and accurately identified spectral differences introduced by artificial colorants, even at low adulteration concentrations (0.1 g.kg^−1^). Notably, SMAFNet also exhibited strong transferability, maintaining high accuracy across multiple colorant-adulterated datasets.

Despite these advancements, some limitations remain. In real-world food safety monitoring, illegally adulterated samples are much rarer than authentic samples, resulting in small and highly imbalanced datasets. This data scarcity poses a significant challenge for SMAFNet as deep learning models typically require abundant and balanced training data to achieve optimal generalization. Although this study focused on detecting individual adulterants in separate experiments to thoroughly evaluate SMAFNet’s performance for each, future research should explore its capability to simultaneously identify multiple colorants. Environmental variability—including fluctuations in humidity and temperature—may also adversely affect the predictive performance of the model, indicating a need for further optimization and rigorous validation to enhance the model’s robustness.

In future work, self-supervised and weakly supervised learning techniques could be integrated to enhance SMAFNet’s ability to detect abnormal adulteration under limited data conditions. Additionally, further validation using broader datasets—including those containing different black tea varieties, samples from different countries or regional markets, and samples contaminated with other types of adulterants—should be conducted to strengthen the model’s generalization and reinforce its practical value in routine food quality control.

## Figures and Tables

**Table 1:** Dataset division for calibration and validation subsets constructed using the SPXY algorithm.

Dataset	Calibration Set	Validation Set
1 (Tartrazine adulteration)	35 (no additive) + 74 (Tartrazine added)	10 (no additive) + 26 (Tartrazine added)
2 (Sunset Yellow adulteration)	37 (no additive) + 72 (Sunset Yellow added)	8 (no additive) + 28 (Sunset Yellow added)
3 (Ponceau 4R adulteration)	39 (no additive) + 70 (Ponceau 4R added)	6 (no additive) + 30 (Ponceau 4R added)

**Table 2:** Performance of the SPM with different kernel sizes and output channel configurations.

Kernel Size	Output Channels	Accuracy (ACC, %)	F1-Score
[8 × 1]	128	68.81	0.7536
	64	68.81	0.7536
[16 × 1]	128	71.56	0.7737
	64	71.56	0.7737
[32 × 1]	128	64.22	0.7068
	64	62.39	0.6870

**Table 3:** Performance of the MSFEM with different kernel sizes and scale combinations.

Kernel and Scale	ACC (%)	F1-Score	Kernel and Scale	ACC (%)	F1-Score	Kernel and Scale	ACC (%)	F1-Score
[3 × 1]	77.98	0.8310	[5 × 1]	79.82	0.8472	[7 × 1]	77.06	0.8299
[1 × 1, 3 × 1]	84.40	0.8874	[3 × 1, 5 × 1]	85.32	0.8873	[7 × 1, 9 × 1]	83.49	0.8816
[1 × 1, 9 × 1]	85.32	0.8947	[5 × 1, 7 × 1]	85.32	0.8857	[9 × 1, 11 × 1]	83.49	0.8816
[1 × 1, 3 × 1, 5 × 1]	88.99	0.9155	[3 × 1, 5 × 1, 7 × 1]	90.83	0.9296	[5 × 1, 7 × 1, 9 × 1]	88.07	0.9139
[1 × 1, 3 × 1, 7 × 1]	89.91	0.9231	[1 × 1, 5 × 1, 9 × 1]	92.66	0.9452	[3 × 1, 7 × 1, 11 × 1]	88.07	0.9128

**Table 4:** Performance of the MSFEM with different depth values.

Depth (d)	ACC (%)	F1-Score
1	92.66	0.9452
2	95.41	0.9660
3	94.50	0.9595

**Table 5:** Performance evaluation of different models on the Sunset Yellow-adulterated dataset.

Model	ACC (%)	Precision (PRE, %)	Recall (REC, %)	F1-Score
PLS-DA	72.22	84.62	78.57	0.8148
RBF-SVM	86.11	89.66	92.86	0.9123
RF	83.33	86.67	92.86	0.8966
1D-CNN	80.56	92.00	82.14	0.8679
MLP	77.78	88.46	82.14	0.8519
SMAFNet	97.22	96.55	100.00	0.9825

**Table 6:** Performance evaluation of different models on the Tartrazine-adulterated dataset.

Model	ACC (%)	PRE (%)	REC (%)	F1-Score
PLS-DA	75.00	79.31	88.46	0.8364
RBF-SVM	88.89	92.31	92.31	0.9231
RF	86.11	86.21	96.15	0.9091
1D-CNN	80.56	92.00	82.14	0.8679
MLP	80.56	80.65	96.15	0.8772
SMAFNet	97.22	96.30	100.00	0.9811

**Table 7:** Performance evaluation of different models on the Ponceau 4R-adulterated dataset.

Model	ACC (%)	PRE (%)	REC (%)	F1-Score
PLS-DA	77.78	88.46	82.14	0.8519
RBF-SVM	83.33	89.29	89.29	0.8929
RF	86.11	89.66	92.86	0.9123
1D-CNN	83.33	89.29	89.29	0.8929
MLP	80.56	86.21	89.29	0.8772
SMAFNet	100.00	100.00	100.00	1

## Data Availability

The original contributions of this study are presented in this article/Supplementary Materials. Further inquiries can be directed to the corresponding authors.

## Supplementary Materials

The following supporting information can be downloaded at: https://www.mdpi.com/article/10.3390/foods14244261/s1, Figure S1. NIR spectra of tablet samples; Figure S2. Confusion matrix on the public dataset; Table S1: The structural parameters of final SMAFNet; Table S2: The validation results of different models on public dataset.

## Abbreviations

The following abbreviations are used in this manuscript:

NIR	Near-infrared
MS-NIR	Mid- and short-wave near-infrared
1D-CNN	One-dimensional convolutional neural network
SMAFNet	Spectral Multi-Scale Attention Fusion Network
SPM	Spectral preprocessing module
MSFEM	Multi-scale feature extraction module
CSAFM	Cross-scale attention fusion module
FEB	Feature extraction block
ReLU	Rectified linear unit
SE	Squeeze-and-excitation
BiFPN	Bidirectional feature pyramid network
ACC	Accuracy
PRE	Precision
REC	Recall
Conv1d	One-dimensional convolutional
PLS-DA	Partial least squares-discriminant analysis
RF	Random forest
MLP	Multilayer perceptron
RBF-SVM	Radial basis function support vector machine
