# Supplementary material for: Spectral Multi-Scale Attention Fusion Network for Rapid Detection of Black Tea Adulteration Using a Handheld Spectrometer
Source: Foods. 2025 Dec 10;14(24):4261. doi: 10.3390/foods14244261 (PMC12732540; doi:10.3390/foods14244261)
Supplement: Supplementary file 1 [file foods-14-04261-s001.zip › foods-3985361-supplementary.pdf]

Supplementary materials

Spectral Multi-Scale Attention Fusion Network for Rapid Detection of Black Tea Adulteration Using a Handheld Spectrometer

Jiawei Tang [1,2,†], Yongyan Chen [1,†], Qing Meng [3,*], Bo Zhao [4,5], Dongling Qiao [1], Guohua Zhao [6] and Jia Chen [1,*]

[1] Chinese-Hungarian Cooperative Research Centre for Food Science, College of Food Science, Southwest University, Chongqing 400715, China; toukai3216@outlook.com (J.T.); cyy040215@outlook.com(Y.C.); qdttkl@163.com (D.Q.)
[2] School of Westa, Southwest University, Chongqing 400715, China
[3] Integrative Science Center of Germplasm Creation in Western China (Chong Qing) Science City, College of Food Science, Southwest University, Chongqing, 400715, China
[4] Key Laboratory of Condiment Supervision Technology, State Administration for Market Regulation, Chongqing 400731, China; zhaobocq@163.com
[5] Chongqing Institute for Food and Drug Control, Chongqing 400731, China
[6] College of Life Sciences, Sichuan Normal University, Chengdu 610101, China; zhaoguohua1971@163.com
* Correspondence: mengqing2015@swu.edu.cn (Q.M.); swaumyh@swu.edu.cn (J.C.)
† These authors contributed equally to this work.

Table S1 The structural parameters of final SMAFNet

**Module**[a]	Composition[b]	Parameter	Input Size	Output Size
**SPM**	Conv1d	kernel=16,stride=2, padding=21	B×1×228	B×64×128
	Max-Pool1	kernel=2,stride=2	B×64×128	B×64×64
**FEB 1-1**	1×1Conv1d	kernel=1,stride=1, padding=0	B×64×64	B×128×64
	Relu		B×128×64	B×128×64
	SE		B×128×64	B×128×64
	Max-Pool	kernel=2,stride=2	B×128×64	B×128×32
**FEB 1-2**	1×1Conv1d	kernel=1,stride=1,padding=0	B×128×32	B×256×32
	Relu		B×256×32	B×256×32
	SE		B×256×32	B×256×32
	Max-Pool	kernel=2,stride=2	B×256×32	B×256×16
**FEB 2-1**	5×1Conv1d	kernel=5,stride=1,padding=3	B×64×64	B×128×64
	Relu		B×128×64	B×128×64
	SE		B×128×64	B×128×64
	Max-Pool	kernel=2,stride=2	B×128×64	B×128×32
**FEB 2-2**	5×1Conv1d	kernel=5,stride=1,padding=3	B×128×32	B×256×32
	Relu		B×256×32	B×256×32
	SE		B×256×32	B×256×32
	Max-Pool	kernel=2,stride=2	B×256×32	B×256×16
**FEB 3-1**	9×1Conv1d	kernel=9,stride=1,padding=5	B×64×64	B×128×64
	Relu		B×128×64	B×128×64
	SE		B×128×64	B×128×64
	Max-Pool	kernel=2,stride=2	B×128×64	B×128×32
**FEB 3-2**	9×1Conv1d	kernel=9,stride=1,padding=5	B×128×32	B×256×32
	Relu		B×256×32	B×256×32
	SE		B×256×32	B×256×32
	Max-Pool	kernel=2,stride=2	B×256×32	B×256×16

[a] The functional module of SMAFNet, including the Spectral Preprocessing Module (SPM) and Feature Extraction Blocks (FEBs) with different convolution kernel sizes (1×1, 5×1, 9×1).

[b] The component(s) of each module, such as 1D convolutional layer (Conv1d), max-pooling layer (Max-Pool), rectified linear unit (ReLU), and Squeeze-and-Excitation (SE) module.

[c] Details key configurations of components, for Conv1d, it includes kernel size, stride, and padding; for Max-Pool, it includes kernel size and stride. No additional parameters are listed for ReLU and SE modules as they use default settings.

[e] and [f]: The dimension of feature tensors in the format "B×C×L", where B is the batch size, C is the number of feature channels, and L is the length of the spectral feature sequence.

.

The tablet dataset is available open-source on https://www.models.life.ku.dk. The NIR spectra of tablet samples are shown in Figure S1. To evaluate the transferability of SMAFNe, RBF-SVM and 1D-CNN were selected as baseline models. The models were evaluated using 10-fold cross-validation, and their performance was assessed based on the average values of accuracy (ACC), precision (PRE), recall (REC), and F1-score. The results are summarized in Table S2, and the prediction confusion matrices for each model are presented in Figure S2.

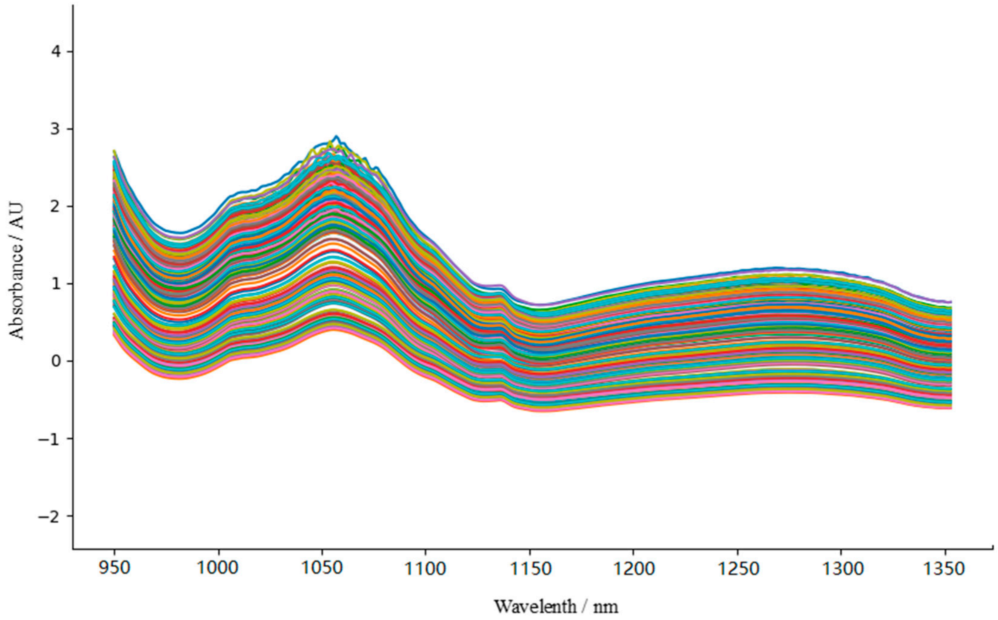

Figure S1. NIR spectra of tablet samples

Table S2. The validation results of different models on public dataset.

Model	ACC (%)	PRE (%)	REC (%)	F1-score
SVM	95.16±3.13	95.91±2.71	95.13±3.19	0.9552±0.0295
1D-CNN	96.45±2.82	96.77±2.63	96.47±2.84	0.9662±0.0274
SMAFNet	98.71±1.67	98.85±1.48	98.75±1.61	0.9880±0.0154

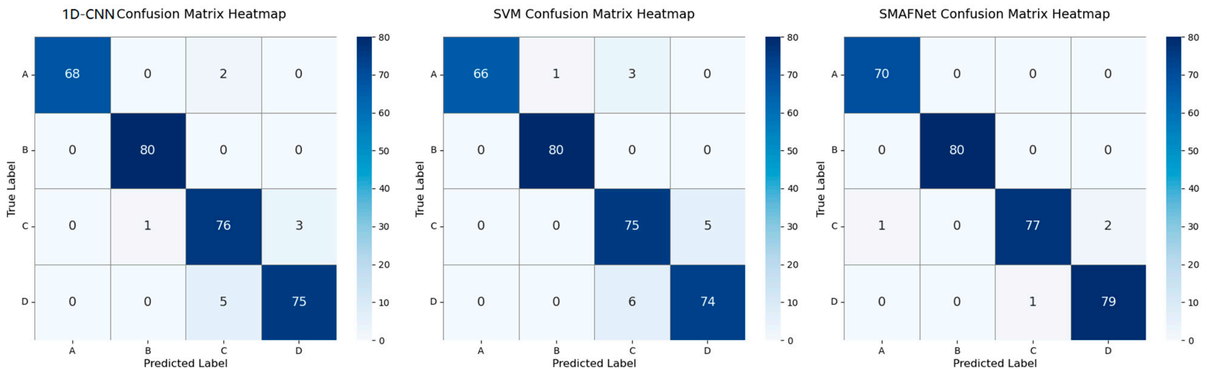

(a) 1D-CNN (b) rbf-SVM (c) SMAFNet

Figure S2. Confusion matrix on the public dataset
